# Alpha 1 Antitrypsin-Deficient Macrophages Have Impaired Efferocytosis of Apoptotic Neutrophils

**DOI:** 10.3389/fimmu.2020.574410

**Published:** 2020-11-20

**Authors:** Jungnam Lee, Yuanqing Lu, Regina Oshins, Jesse West, Craig G. Moneypenny, Kyudong Han, Mark L. Brantly

**Affiliations:** ^1^ Division of Pulmonary, Critical Care and Sleep Medicine, University of Florida, Gainesville, FL, United States; ^2^ Department of Microbiology, College of Science and Technology, Dankook University, Cheonan, South Korea; ^3^ DKU-Theragen Institute for NGS Analysis, Cheonan, South Korea

**Keywords:** Alpha 1 antitrysin, AAT deficiency, neutrophil, macrophage, efferocytosis, cytokine

## Abstract

Alpha 1 antitrypsin deficiency (AATD) is an autosomal co-dominant disorder characterized by a low level of circulating AAT, which significantly reduces protection for the lower airways against proteolytic burden caused by neutrophils. Neutrophils, which are terminally differentiated innate immune cells and play a critical role to clear pathogens, accumulate excessively in the lung of AATD individuals. The neutrophil burden in AATD individuals increases the risk for early-onset destructive lung diseases by producing neutrophil products such as reactive oxygen radicals and various proteases. The level of AAT in AATD individuals is not sufficient to inhibit the activity of neutrophil chemotactic factors such as CXCL-8 and LTB4, which could lead to alveolar neutrophil accumulation in AATD individuals. However, as neutrophils have a short lifespan, and apoptotic neutrophils are rapidly cleared by alveolar macrophages that outnumber the apoptotic neutrophils in the pulmonary alveolus, the increased chemotaxis activity does not fully explain the persistent neutrophil accumulation and the resulting chronic inflammation in AATD individuals. Here, we propose that the ability of alveolar macrophages to clear apoptotic neutrophils is impaired in AATD individuals and it could be the main driver to cause neutrophil accumulation in their lung. This study demonstrates that Z-AAT variant significantly increases the expression of pro-inflammatory cytokines including CXCL-8, CXCL1, LTB4, and TNFα in LPS-treated macrophages. These cytokines play a central role in neutrophil recruitment to the lung and in clearance of apoptotic neutrophils by macrophages. Our result shows that LPS treatment significantly reduces the efferocytosis ability of macrophages with the Z-AAT allele by inducing TNFα expression. We incubated monocyte-derived macrophages (MDMs) with apoptotic neutrophils and found that after 3 h of co-incubation, the expression level of CXCL-8 is reduced in M-MDMs but increased in Z-MDMs. This result shows that the expression of inflammatory cytokines could be increased by impaired efferocytosis. It indicates that the efferocytosis ability of macrophages plays an important role in regulating cytokine expression and resolving inflammation. Findings from this study would help us better understand the multifaceted effect of AAT on regulating neutrophil balance in the lung and the underlying mechanisms.

## Introduction

Alpha-1-antitrypsin (AAT) is a protease inhibitor that regulates the proteolytic effects of neutrophil-derived serine proteases, including neutrophil elastase, cathepsin G, and thrombin (1, 2). It is produced mainly by hepatocytes but also by monocytes, macrophages, and bronchial cells (3). AAT is a classical acute phase response protein and its serum level is increased during states of acute inflammation (2). AATD results from mutations in the *SERPINA1* gene. Approximately 120 variant alleles of AAT have been reported to date. The most common allele is M, and Z is a mutated allele most commonly responsible for severe deficiency. It is characterized by a single amino acid substitution of lysine for glutamic acid at position 342, leading to conformational change to its latent form or polymerization, lowering the concentration of circulating AAT. Therefore, AAT does not reach lung tissues where it normally acts as the primary regulator against proteolytic activities in AATD individuals (4). AATD predisposes individuals to lung diseases, including chronic obstructive pulmonary disease (COPD) (5, 6). AATD is indeed responsible for 1%–2% of COPD, and AATD-associated lung disease shares major features of emphysema (7).

AATD individuals have a higher number of neutrophils in their lower respiratory tract than healthy normal individuals (8–10). Neutrophils could be considered a double-edged sword. They are important mediators of host defenses, typically being the first leukocytes recruited to an inflammatory site and eliminating pathogens to resolve the inflammation. However, uncontrolled neutrophilic activity and continued neutrophil recruitment to inflammatory sites can result in an excess of reactive oxygen radicals and various proteolytic enzymes that could cause damage to the surrounding healthy lung tissues and persistent inflammation, a feature of many human diseases including COPD and cystic fibrosis (11–13). Excessive numbers of neutrophils have been implicated in the pathogenesis of many acute and chronic lung diseases (14). Thus finding molecular mechanisms responsible for alveolar neutrophil accumulation in AATD individuals is important to understand risks of lung diseases in AATD individuals.

Alveolar macrophages reside at the interphase between air and lung tissue, serving as the front line of cellular defense against respiratory pathogens. They are the primary phagocytes of the innate immune system, clearing the lower respiratory tract of allergens and infectious or toxic particles (15). When faced with infectious particles or microbes, alveolar macrophages produce pro-inflammatory cytokines to initiate inflammatory responses and recruit neutrophils into the alveolar spaces (16). Neutrophils migrate out of the pulmonary capillaries into the air spaces to serve as the second-line defense. After the phagocytosis of infectious microbes, neutrophils undergo programmed cell death and are cleared by alveolar macrophages, orchestrating the resolution of inflammation and tissue repair (17). However, if apoptotic neutrophils are not efficiently cleared by alveolar macrophages, the apoptotic cells release potentially injurious cytoplasmic contents into the alveolus, causing further tissue injury and perpetuating inflammation (18, 19).

Neutrophil accumulation is considered to be the main source of proteolytic burden in the lung of AATD individuals, and airway neutrophilic inflammation is closely linked to tissue destruction and alveolar airspace enlargement, leading to disease progression (20, 21). Nonetheless, the role of AAT in maintaining alveolar neutrophil homeostasis has not been fully examined. In this study, we hypothesized that Z-AAT impairs neutrophil homeostasis in pulmonary alveoli by increasing the expression of neutrophil chemotactic factors and by decreasing the ability of macrophages to clear apoptotic cells. We compared the expression level of pro-inflammatory cytokines between lipopolysaccharide (LPS)-treated M- and Z-monocyte derived macrophages (MDMs). The cytokines examined in this study include potent neutrophil chemotactic factors chemokine (C-X-C motif) ligand 1 (CXCL-1) and ligand 8 (CXCL-8) (22, 23). Due to difficulty obtaining primary human alveolar macrophages, for the present study we used macrophages derived from monocytes (MDM). The macrophages were maturated in the presence of macrophage colony-stimulating factor (M-CSF) and granulocyte-macrophage colony-stimulating factor (GM-CSF) as previously described (24). MDMs from PiMM individuals are referred to as M-MDM while MDMs from PiZZ individuals are referred to as Z-MDM in this study. Efferocytosing macrophages promote resolution of inflammation by suppressing the expression of inflammatory cytokines (25). Therefore macrophage efferocytosis impaired by LPS, which is known to inhibit the efferocytosis of apoptotic neutrophils by macrophages, results in persistent expression of inflammatory cytokines (26). We investigated whether AAT regulates the inhibitory effect of LPS on macrophage efferocytosis. This study demonstrates that Z-AAT reinforces the inhibitory effect of LPS on macrophage efferocytosis by increasing TNFα expression and delays the suppression of CXCL-8 mediated by efferocytosis of apoptotic cells. Our results also show that TNFα could be responsible for the LPS-reduced macrophage efferocytosis by inhibiting the expression of efferocytosis-associated molecules, CD14, CD36, and RARα. Taken together, this study explains the function of Z-AAT to exacerbate neutrophil burden and consequent pulmonary inflammation. Therefore, the findings from this study may translate to identifying a targeted strategy to control alveolar neutrophil balance in AATD individuals.

## Materials and Methods

### Monocyte Isolation and Macrophage Differentiation

Using ficoll-gradient centrifugation, peripheral blood mononuclear cells were isolated from the blood of outpatient volunteers (University of Florida Institutional Review Board protocol 2015-01051). Characteristics of the volunteers are shown in **Table 1**. Monocytes were purified using a monocyte enrichment kit (Stemcell Technology, Vancouver), following the manufacturer’s instruction. Monocytes were plated in 12-well plates at 300,000 cells per well in macrophage differentiation media (RPMI 1640 containing 10% heat-inactivated FBS, 20 Units/ml penicillin, 20 μg/ml streptomycin, 250 ng/ml Amphotericin B, recombinant human GM-CSF (0.5 ng/ml) and recombinant human M-CSF (5 ng/ml)) and differentiated for 7 days. Both growth factors, GM- and M-CSF, exist in the lung, and especially GM-CSF is important to induce AAT expression (24). Supplemental medium (50% of the volume in each well) was added every 3 days after removal of half of the old media, and cells were used on day 7 for any treatment. To induce inflammatory signaling in MDMs, the cells were stimulated with 10 ng/ml LPS from Escherichia coli O111:B4 (Sigma-Aldrich, St. Louis) overnight. Non-treated and LPS-treated MDMs were harvested for RNA extraction using the Qiagen RNeasy kit (Qiagen, Hilden). To inhibit LPS-mediated TNFα signaling, MDMs were pretreated with LPS for 1 h and incubated with TNFα neutralizing antibody (MAB210, R&D Systems, Minneapolis) or isotype control (MAB002, R&D Systems, Minneapolis) for 18 h.

**Table 1:** Characteristics of controls and AATD individuals.

Characteristic	PiMM (n=6)	PiZZ (n=6)	P-value
Age	48.5 (22–68)	51 (39–69)	0.82
Gender (M/F)	4/2	4/2	N/A
FEV1% predicted	86.6 (57–114)	60.8 (23.2–97)	0.14
FEV1/FVC	77.6 (59–91)	55.3 (31.6–71.8)	0.04
Current smoker	No	No	N/A

Definition of abbreviations: PiMM, individuals homozygous for normal PiM allele; PiZZ, individuals homozygous for mutant PiZ allele; N/A, not applicable; FEV1, forced expiratory volume in one second; FVC, forced vital capacity.

### Immunofluorescence

To examine AAT distribution, MDMs were differentiated on glass slides and fixed in 4% paraformaldehyde for 20 min and permeabilized for 10 min in PBS containing 0.01% Triton X-100. The permeabilized cells were incubated with rabbit anti AAT polyclonal antibody (Abcam, Cambridge) at 1:400 dilution in PBS containing 0.1% Tween 20 for 1 h. After washing with PBS-Tween 20, cells were immunostained with Alexa Fluor488 goat anti-rabbit (Abcam, Cambridge) at 1:500 dilution at room temperature for 1 h. The immunostained cells were mounted on glass slides using VECTASHIELD mounting media with DAPI and examined using a fluorescence microscope (BZ-X700, Keyence, Osaka). For quantification of intracellular AAT, AAT fluorescent intensity and number of cells were measured with BZ software, and the fluorescent intensity was normalized to the cell number. To examine CD36 distribution, MDMs were incubated with mouse anti CD36 monoclonal antibody (ThermoFisher, Waltham) at 1:20 dilution in blocking solution (Invitrogen, Carlsbad) overnight at 4°C. After washing with PBS-Tween 20, cells were immunostained with Alexa Fluor647 goat anti-rabbit (Abcam, Cambridge) at 1:1000 dilution at room temperature for 1 h. For quantification of CD36, ~1000 MDMs were evaluated for each MDM group (n=4).

### Western Blot Analysis

Total proteins were extracted from MDMs using RIPA lysis buffer (Cell Signaling, Danvers) plus protease and phosphatase inhibitors. The protein concentration of each sample was measured using a standard Bradford assay (BioRad, Hercules) and equal amounts of protein were loaded onto a 12% SDS polyacrylamide gel. After gel electrophoresis, the proteins were transferred onto a nitrocellulose membrane using a wet-transfer system, and the membrane was blocked in Tris-buffered saline with 0.1% Tween 20 (TBST) containing 5% nonfat dry milk. The membrane was immunoblotted overnight at 4°C with AAT rabbit polyclonal antibody (DAKO, Carpinteria) at a dilution of 1:5,000 in TBST. Horseradish peroxidase conjugated anti-rabbit antibody (BioRad, Hercules) was used for secondary labeling at 1:5,000 in TBST for 1 h at room temperature. The membrane was reprobed with GAPDH rabbit polyclonal antibody (Proteintech, Rosemont) at 1:5,000 in TBST. A horseradish peroxidase conjugated anti-rabbit (BioRad, Hercules) was used for secondary labeling. Protein bands were visualized by enhanced chemiluminescence (ECL, GE Healthcare, Chicago).

### ELISA

AAT was measured in conditioned media of MDMs using a sandwich enzyme-linked immunosorbent assay (ELISA). Ninety-six well ELISA plates were coated with goat anti-human AAT antibody at 4°C overnight. The wells were blocked with PBS containing 0.05% Tween 20 and 0.5% BSA for 1 h at room temperature. Control and samples were added to the ELISA plate and incubated for 2 h at 37°C. After the ELISA plates were washed, bound AAT remained in each well. Rabbit anti-human AAT antibody (DAKO, Carpinteria) was added to the plate and incubated for 1 h at 37°C, followed by HRP-conjugated goat anti-mouse antibody (Bio-Rad, Hercules). After washing, QuantaBlu Fluorogenic Peroxidase Substrate (ThermoFisher, Waltham) was added to the wells and incubated for 5 min at room temperature. HRP activity was read at an excitation/emission maxima of 325/420 using a spectrophotometer (LS50B LuminSpectrometer, Perkin Elmer, Waltham). The concentrations of CXCL-8 (Abcam, Cambridge), LTB4 (Cayman Chemical, Ann Arbor), and TNFα (Abcam, Cambridge) were measured in conditioned media of MDMs by ELISA, following the manufacturer’s instruction.

### Neutrophil Isolation and Chemotaxis Assay

Primary neutrophils were isolated from PiMM volunteers using EasySep direct human neutrophil isolation kit (Stemcell Technologies, Vancouver), following the manufacturer’s instruction. The isolated cells were incubated with fluorescent CD16 and CD66b antibodies (BioLegend, San Diego). Percentage of CD16 and CD66b-positive cells was calculated using a Gallios flow cytometer with Kaluza software (Beckman Coulter, Brea, **Supplementary Figure 1**). Neutrophil chemotaxis was assayed in a Transwell system using polycarbonate membranes with 3-μm pore size (Corning, Corning). MDMs were incubated with or without LPS and, supernatant from the cell culture was transferred into the bottom layer of the chemotaxis chamber. Freshly isolated neutrophils (1 x 10^6^) were added into the top layer of the chamber. The neutrophils migrated from the top to the bottom layer for 30 min. Migrated cells were counted using a hemocytometer and automated cell counter (Invitrogen, Carlsbad).

### AAT Treatment

Lyophilized AAT (Prolastin-C) was reconstituted with deionized water, following the manufacturer’s instruction and stored at -80°C. To examine whether AAT is able to inhibit LPS-mediated CXCL-8 expression, MDMs were incubated with LPS and different concentrations of AAT for 18 h. AAT-treated MDMs were lysed for RNA extraction using the Qiagen RNeasy kit (Qiagen, Hilden), and CXCL-8 expression was examined using qRT-PCR.

### Neutrophil Apoptosis

To induce apoptosis in the cells, neutrophils were aged for 20 h and then incubated with 1 μM staurosporine for another 3 h. The apoptotic rate of the neutrophils was assessed by flow cytometry with Annexin V/propidium iodide staining (Invitrogen, Carlsbad). For efferocytosis assay, the apoptotic neutrophils were labeled with CellTracker Red CMTPX dye (Invitrogen, Carlsbad), following the manufacturer’s instruction.

### Efferocytosis Assay

Following 7 days of macrophage differentiation, MDMs were incubated with or without LPS for 18 h, and then the MDMs were incubated with CellTracker Red CMTPX dye-labeled apoptotic neutrophils suspended at 1 x 10^6^/ml at 37°C for 30 min, providing a phagocyte to target ratio of 1:4. After the incubation, the non-ingested neutrophils were removed by repeated washing with PBS. Removal of the non-ingested neutrophils was confirmed with light microscope (Olympus 1X70), and phagocytosis of the neutrophils by MDMs was confirmed by fluorescence microscopy (BZ-X700, **Supplementary Figure 2**). MDMs were incubated with Accutase (Stemcell Technologies, Vancouver) at room temperature for 20 min, followed by 15 min on ice. After the incubation with Accutase, MDMs were collected by a gentle scraping with a plastic scraper and analyzed by Gallios flow cytometer with Kaluza software (Beckman Coulter, Brea). A minimum of 10,000 events was acquired per sample. For each MDM sample, the efferocytosis rate of control MDM was set to 100%.

### Gene Expression Validation by qRT-PCR

Total RNA (1 μg), extracted from MDMs, was reverse-transcribed using SuperScript^®^ VILO Master Mix (Invitrogen, Carlsbad), according to the manufacturer’s instruction. Quantification of PCR products was performed with 7500 Fast Real-time PCR (Applied Biosystems, Foster City). SensiFAST Real-Time PCR Kit (Bioline, London) was used to produce fluorescence-labeled PCR products and to monitor increasing fluorescence during repetitive cycling of the amplification reaction. Taqman probes/primers specific for the CXCL-1, CXCL-8/IL-8, TNFα, CD14, CD36, RARα genes, and for the GNB2L1 gene, as a housekeeping gene, were used in the real-time PCR reaction. Expression levels of the genes were obtained using the classical 2^(-ΔΔCt) method.

### CXCL-8 Suppression by the Efferocytosis of Apoptotic Cells

MDMs were treated with LPS (10 ng/ml) for 30 min and then they were incubated with apoptotic neutrophils or Jurkat cells for 3 h. To induce apoptosis in Jurkat cells, the cells were exposed to UV (200 mJ/cm^2^) in PBS and the UV-treated cells were incubated in RPMI 1640 for 4 h before being incubated with MDMs. Non-ingested apoptotic cells were washed off with PBS five times and MDMs were lysed for RNA isolation. The expression level of CXCL-8 was examined in the RNA samples using qRT-PCR. The efferocytosis-mediated suppression of cytokine levels was compared between M- and Z-MDMs.

### Statistical Analysis

Results are expressed as the mean of number of independent experiments using MDMs from different donors. The assessment was evaluated by two-tailed Student’s t-test, one-way ANOVA test or two-way ANOVA test. Bonferroni test was used for multiple comparisons. P-values of 0.05 or less were considered to be statistically significant.

## Results

### Z-AAT Retained in MDMs

**Figure 1:**
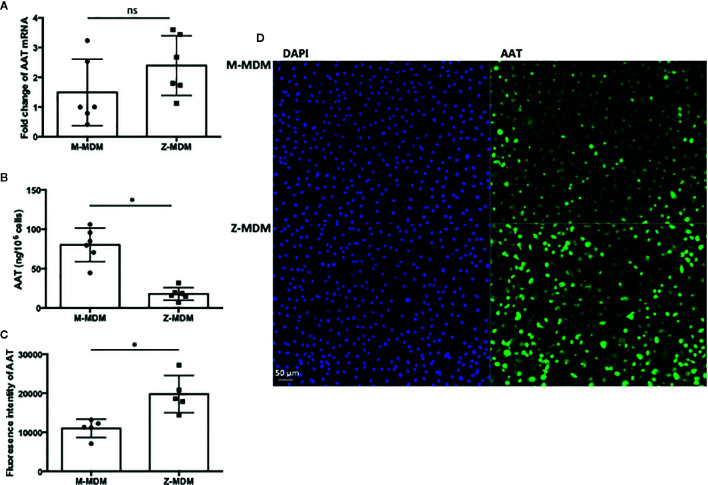
AAT in M- and Z-MDMs. MDMs and their conditioned media were collected at day 7 of macrophage differentiation. AAT mRNA and protein levels are compared in M- and Z-MDMs (n=6). **(A)** The level of AAT mRNA was measured in M- and Z-MDMs using qRT-PCR. **(B)** The concentration of AAT was measured in conditioned media of M- and Z-MDMs using ELISA. **(C, D)** MDMs were immunostained for AAT (green) and the level of intracellular AAT was estimated based on the intensity of positive signal. 20x Images were taken using a fluorescence microscope; bar 50 μm. ~2,000 cells, originating from three separate experiments, were evaluated for each MDM group. *Denotes statistical significance (p-value < 0.05) according to two-tailed Student’s t-test.

AAT mRNA expression level was compared between M- and Z-MDMs. As shown in **Figure 1A**, the gene expression level of AAT was similar between the two groups (p-value=0.17). The concentration of AAT was also compared in conditioned media of M- and Z-MDMs, and the result shows that the AAT concentration is significantly higher in the media of M-MDMs than that of Z-MDMs (**Figure 1B**, p-value<0.0001), indicating a higher secretion rate of M-AAT than Z-AAT. MDMs were immunostained for AAT (**Figure 1C**), and the level of intracellular AAT was quantified in the cells. The level of intracellular AAT was significantly higher in Z-MDMs than M-MDMs (**Figure 1D**, p-value=0.0006).

### Neutrophil Chemoattractant Production by LPS-Stimulated MDMs

**Figure 2:**
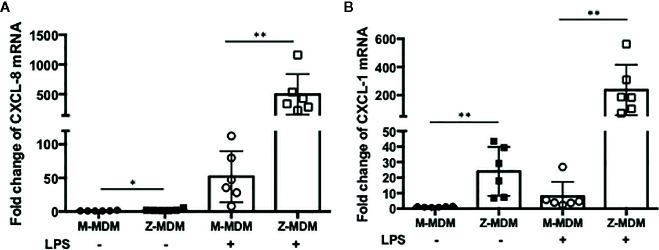
LPS-induced neutrophil chemoattractant expression in M- and Z-MDMs. MDMs were incubated with LPS (10 ng/ml) overnight and the expression levels of **(A)** CXCL-8 and **(B)** CXCL-1 were compared between M- and Z-MDMs (n=6). The expression levels of the cytokines were normalized to GNB2L1, housekeeping gene.* and **Denote statistical significance (p-value < 0.05 and p-value < 0.01, respectively) according to two-tailed Student’s t-test.

**Figure 3:**
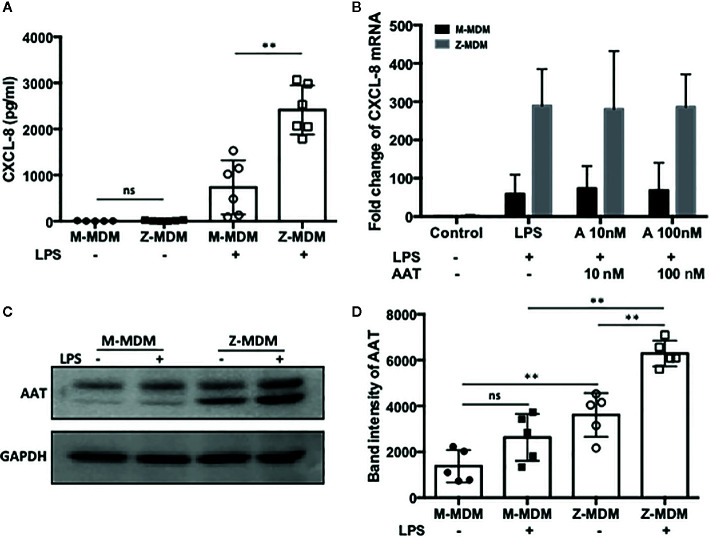
A high level of CXCL-8 resulted from Z-AAT accumulation. MDMs were incubated with LPS (10 ng/ml) overnight. **(A)** CXCL-8 protein level was measured and compared between LPS-treated M- and Z-MDMs (n=6). **(B)** Two different concentrations of AAT were extracellularly added to M- and Z-MDM cultures and the cells were incubated with LPS and AAT overnight. Two-way ANOVA test was used to analyze the effect of genotype and treatment (AAT) on the expression of CXCL8 in the cells (n=5). P-values of interaction, genotype, and treatment were 0.96, 0.0001, and 0.996, respectively. *Denotes statistical significance (p-value < 0.05) according to two-tailed Student’s t-test. **(C, D)** Total proteins were collected from control and LPS-treated MDMs and the increased intracellular AAT levels in LPS-treated cells were visualized and quantified using western blotting (n=5). One-way ANOVA was used to compare the level of intracellular among the samples, and p-value was less than 0.0001. **Denotes statistical significance (p-value < 0.01) according to one-way ANOVA multiple comparisons.

The innate immune response usually begins with the activation of alveolar macrophages to produce various cytokines, including neutrophil chemoattractants in the lung, and chemotaxis by neutrophils plays a critical role in the innate immune response. CXCL-8 and CXCL-1 are potent neutrophil chemoattractants on an equimolar basis (27–29). The concentration of AAT is about five-fold higher in conditioned media of M-MDM compared with Z-MDM (24). To examine the effect of AAT on the expression of CXCL-8 and CXCL-1, their expressions were compared between LPS-treated M- and Z-MDMs. LPS remarkably increased the expression of the two neutrophil chemoattractants in both M- and Z-MDMs (**Figures 2A, B**). The expression level of CXCL-8 was increased ~200-fold by LPS in Z-MDMs, and CXCL-1 expression was increased ~40-fold by LPS treatment in Z-MDMs. The expression level of CXCL-8 and CXCL-1 was significantly higher in LPS-treated Z-MDMs than M-MDMs (p-value < 0.05 for the comparison). It was previously reported that the level of CXCL-8 is significant higher in bronchoalveolar lavage fluid of AATD individuals than healthy controls (30). We measured the concentrations of CXCL-8 in conditioned media of LPS-treated MDMs using ELISA. The result shows that CXCL-8 concentration is significantly higher in conditioned media of LPS-treated Z-MDMs than M-MDMs (**Figures 3A**, p-value=0.0004), consistent with the previous finding. The higher expression level of CXCL-8 in Z-MDMs could result from lower concentration of extracellular AAT, higher level of accumulated intracellular AAT, or both. Exogenous AAT was added to M- and Z-MDM cultures to give a similar concentration of extracellular AAT between the two MDM cultures to eliminate the effect caused by different extracellular AAT concentrations on CXCL-8 expression between the cells. The result shows that even when the concentration of extracellular AAT is very similar between the two MDM cultures, the expression level of CXCL-8 is still significantly higher in Z-MDMs compared with M-MDMs (**Figure 3B**). It indicates that intracellular Z-AAT led to the higher expression of CXCL-8 in LPS-treated Z-MDMs. As AAT is an acute phase protein, its expression level is increased by LPS treatment, leading to a higher level of intracellular Z-AAT in the cells (**Figure 3C**). The level of intracellular AAT was quantified and compared between controls and LPS-treated MDMs. As shown in **Figure 3D**, the intracellular AAT level was not statistically different between M-MDM controls and LPS-treated M-MDMs, but it was significantly higher in LPS-treated Z-MDMs than their controls (p-value<0.0003). To examine whether AAT expression level is correlated with the expression of CXCL-8 in Z-MDMs, we calculated a Pearson’s correlation coefficient and p-value between the expression levels of the two genes. The result shows that the expression of AAT is highly correlated with the expression of CXCL-8 in Z-MDMs (**Table 2**).

**Table 2:** Correlation between the expression levels of AAT and CXCL-8.

Z-MDM culture condition	n	AAT	CXCL-8	Pearson r	P-value
Control	5	1.0	1.0		
LPS (10 ng/ml)	5	2.7	289.4		
LPS (10 ng/ml)+AAT (10 nM)	5	2.8	280.4		
LPS (10 ng/ml)+AAT (100 nM)	5	3.4	286.2		
LPS (10 ng/ml)+AAT (1 μM)	5	2.6	171.9		
LPS (10 ng/ml)+AAT (10 μM)	5	2.9	164.5	0.883	0.02

We also examined the level of leukotriene B4 (LTB4), a well-known neutrophil chemoattractant, in conditioned media of M- and Z-MDMs using ELISA. The concentration of LTB4 was below the lower limit of detection level in the media of M-MDM samples while it was detected in Z-MDM samples. The concentration of LTB4 was on average 93.6 ± 53.7 pg/ml in Z-MDM samples. The release of LTB4 is stimulated when alveolar macrophages are exposed to neutrophil elastases (10). It could explain the low level of LTB4 in conditioned media of the MDM cultures.

### Neutrophil Chemotaxis Increased in Z-MDMs

**Figure 4:**
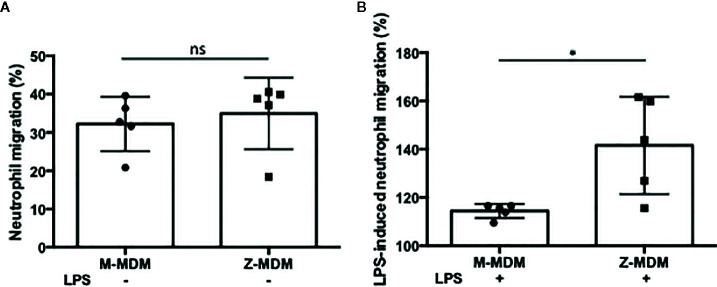
The effect of AAT on LPS-induced neutrophil chemotaxis. MDMs were incubated with LPS (10 ng/ml) overnight. To examine the ability of AAT to regulate neutrophil transmigration, conditioned media of M- and Z-MDMs were collected and placed in the bottom chamber of transwell system. Freshly isolated neutrophils were placed in the top chamber of the transwell system. The number of neutrophils migrated to the bottom layer were counted after 30 min. **(A)** The number of neutrophils in the bottom layer was divided by the total number of neutrophils to calculate the migration rate of control MDMs. **(B)** To calculate the migration rate after LPS treatment, the migration rate of control MDM was set to 100%, and the migration rate of LPS-treated MDM was normalized to control for each individual; n=5. *Denotes statistical significance (p-value < 0.05) according to two-tailed Student’s t-test.

AATD individuals have a higher number of alveolar neutrophils than non-AATD individuals (9, 31, 32). We suspected that the higher expression of the neutrophil chemoattractant factors in LPS-stimulated Z-MDMs could lead to higher neutrophil migration. We freshly isolated neutrophils from human blood and immediately used them for a neutrophil chemotaxis assay because their half-life is short, generally 6–8 h (33). We compared neutrophil migration rates between control and LPS-treated MDMs. Neutrophil migration rate was higher in the conditioned media of LPS-treated MDMs than that of non-treated controls, and the neutrophil migration rate of non-treated controls are shown in the **Figure 4A**. We calculated the neutrophil migration rate increased by LPS treatment in each MDM group. As shown in **Figure 4B**, LPS-mediated increase in the neutrophil migration rate was significantly higher in conditioned media of Z-MDMs than M-MDMs (p-value=0.039). This result correlates with our previous observation that the expression level of neutrophil chemoattractant factors was significantly higher in Z-MDMs than M-MDMs. To confirm that the neutrophils were migrated by chemotaxis other than chemokinesis, three different solutions of PBS, RPMI 1640, and macrophage-differentiated media were used as negative controls for the neutrophil migration assay. The result showed that random neutrophil migration rates in the three solutions were less than 3%, supporting that the observed neutrophil migration in conditioned media of MDMs was mediated by neutrophil chemoattractant factors produced by macrophages.

### Efferocytosis of Apoptotic Neutrophils Reduced by LPS

**Figure 5:**
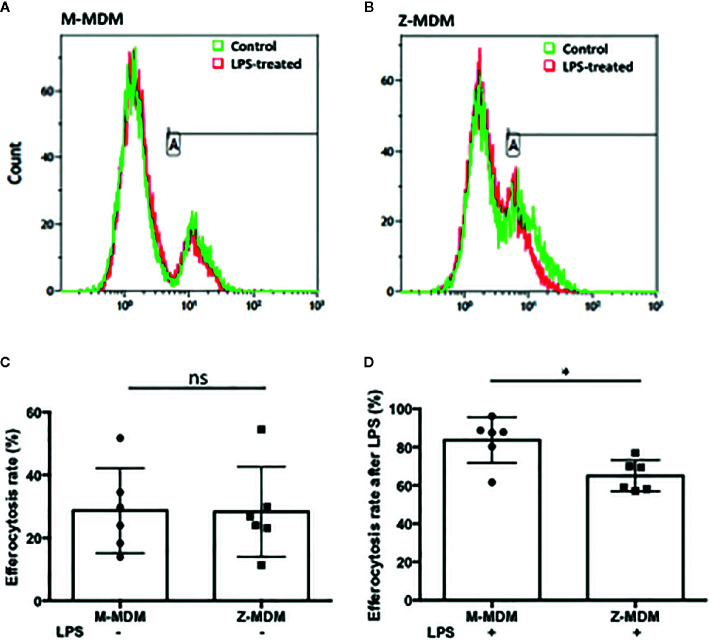
Efferocytosis of apoptotic neutrophils by M- and Z-MDMs. MDMs were incubated with LPS (10 ng/ml) overnight and celltracker red-labeled apoptotic neutrophils were added to the MDM culture. Efferocytosis of the apoptotic cells by MDMs was assessed by flow cytometric analysis **(A)** M-MDM **(B)** Z-MDM. In each histogram plot, the green and red graphs indicate control and LPS-treated MDMs, respectively. The first peak of the each graph indicates non-phagocytosing MDM population and the second peak indicates phagocytosing MDM population. **(C)** The efferocytosis rates of MDM controls were assessed by flow cytometric analysis; n=6. **(D)** Efferocytosis rates of LPS-treated MDMs are expressed relative to those of MDM controls that are set to 100%; n=6. *Denotes statistical significance (p-value < 0.05) according to two-tailed Student’s t-test.

**Figure 6:**
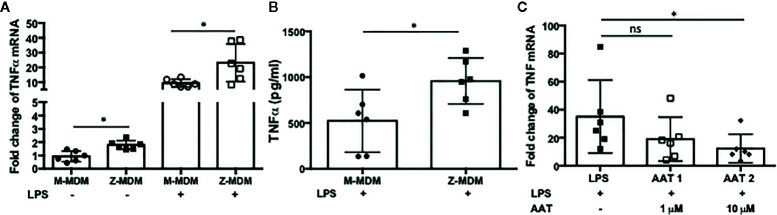
LPS-induced TNFα expression in M- and Z-MDMs. MDMs were incubated with LPS (10 ng/ml) overnight and the gene expression level of **(A)** TNFα and **(B)** its protein level were compared between M-MDMs and Z-MDMs. **(C)** Z-MDMs were incubated with LPS (10 ng/ml) and two different concentrations of M-AAT overnight. The effect of M-AAT on the expression of TNFα was examined in the cells; n=6. *Denotes statistical significance (p-value < 0.05) according to two-tailed Student’s t-test.

**Figure 7:**
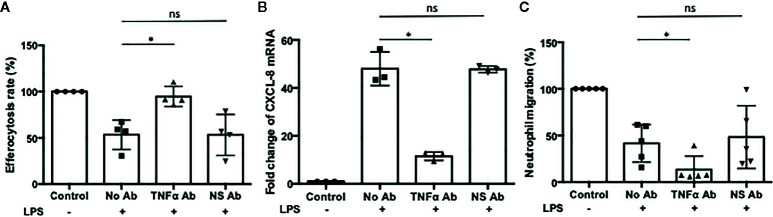
The effect of TNFα on macrophage efferocytosis and neutrophil chemotaxis. Z-MDMs were incubated with LPS (10 ng/ml) and TNFα neutralizing antibody overnight. **(A)** The effect of TNFα neutralizing antibody on neutrophil efferocytosis by MDMs was examined (n=4). For each Z-MDM individual sample, the efferocytosis rate of control MDM was set to 100%, and the efferocytosis rates of the other three treatments were normalized to control MDM. **(B)** The relative expression levels of CXCL-8 in the LPS-treated cells were calculated in comparison to their non-treated controls (n=3). The CXCL-8 expression level of control MDM was set to 1, and the CXCL-8 expression levels of the other three treatments were normalized to control MDM. **(C)** Conditioned media of Z-MDM samples were collected for neutrophil chemotaxis assay (n=5). The number of migrated neutrophils was counted, and neutrophil migration rate was calculated based on the number. The migration rate of control MDM was set to 100%, and the migration rates of the other three treatments were normalized to control MDM. *Denotes statistical significance (p-value < 0.05) according to two-tailed Student’s t-test.

Neutrophils recruited to the site of infection phagocytize bacteria and the process typically accelerates neutrophil apoptosis. Alveolar macrophages clear the apoptotic neutrophils, which ultimately promotes resolution of the bacterial infection (34). Thus the ability of alveolar macrophages to phagocytize apoptotic cells in a timely manner is critical to orchestrate the resolution of inflammation. To determine the ability of M- and Z-MDMs to efferocytose apoptotic neutrophils, we performed efferocytosis of apoptotic neutrophils by MDMs in the presence or absence of LPS. The efferocytosis rate was on average similar between the two MDM groups in the absence of LPS (**Table 3** and **Supplementary Figure 2D**). It was previously reported that LPS inhibits efferocytosis of apoptotic neutrophils by MDMs (26). We compared the reduced efferocytosis rate caused by LPS treatment between M- and Z-MDMs. The result showed that the inhibitory effect of LPS was significantly higher in Z-MDMs than M-MDMs (**Figures 5A–D** (p-value=0.01)). We compared the expression of macrophage efferocytosis-related genes in LPS-stimulated M- and Z-MDMs. The examined molecules were PPARα, PPARγ, CEBPβ, ADAM17, and TNFα. These genes have previously been reported to be involved in efferocytosis. However, unlike the expression of TNFα, the expression levels of all other genes were not statistically different between M- and Z-MDMs (data not shown). LPS stimulation increased the expression of TNFα in both M-and Z-MDMs, but TNFα expression level was significantly higher in Z-MDMs (**Figure 6A**, p-value=0.027). The protein level of TNFα was also significantly higher in conditioned media of LPS-treated Z-MDMs than that of M-MDMs (**Figure 6B**, p-value=0.032). To investigate if extracellular AAT is able to inhibit LPS-induced TNFα expression in Z-MDMs, we incubated LPS-stimulated MDMs with two different concentrations, 1 and 10 μM, of M-AAT, and examined TNFα expression in the cells. The result shows that only a higher concentration of AAT, which is similar to the AAT concentration found in circulating blood of non-AATD individuals, could suppress TNFα expression in LPS-stimulated Z-MDMs, as shown in **Figure 6C** (p-value=0.018). It supports that AAT could inhibit LPS-mediated TNFα expression in macrophages. The ability of AAT to inhibit TNFα expression in human neutrophils was previously reported (35). We suspected that different TNFα expression levels between M-and Z-MDMs could be responsible for the different efferocytosis rates observed between the cells. In order for TNFα to exert its biological function, it has to bind to its specific receptors. To prevent TNFα from binding to its receptors, we incubated Z-MDMs with TNFα neutralizing antibodies. We then examined whether blocking TNFα signaling could abolish the inhibitory effect of LPS on the efferocytosis of apoptotic neutrophils by MDMs. The inhibitory effect of LPS on macrophage efferocytosis was significantly reduced when MDMs were incubated with the neutralizing antibody to TNFα (**Figure 7A**, p-value=0.011). LPS reduced efferocytosis rate by 53% in Z-MDMs but the efferocytosis rate was recovered to 94% by TNFα neutralizing antibody in the cells. The result proposes that LPS inhibits macrophage efferocytosis through TNFα in the cells. The efferocytosis rate of the MDMs that were incubated with a combination of LPS and non-specific isotype antibody was similar to that of the MDMs incubated with LPS alone.

**Table 3:** The efferocytosis rate of M- and Z-MDMs.

	Efferocytosis rate (%)	P-value
M-MDM	28.7 ± 13.5	0.959
Z-MDM	28.3 ± 14.3	

### The Expression of Neutrophil Chemoattractant Factors Regulated by TNFα

TNFα is known to self-regulate its own expression by activating NF-kB signaling (36–38). Regarding that NF-kB signaling regulates the expression of CXCL-8 and CXCL-1 (39, 40), we suspected that TNFα might be responsible for the increased expression of CXCL-8 and CXCL-1 in LPS-stimulated Z-MDMs. We examined whether their expression is reduced when TNFα signaling is inhibited in Z-MDMs. The expression level of CXCL-8 was significantly reduced when MDMs were incubated with TNFα neutralizing antibody (**Figure 7B**, p-value=0.019). The expression level of CXCL-1 was also reduced in the cells but the degree of the reduction was not significant (data not shown). We then performed neutrophil migration in conditioned media of the Z-MDMs in which TNFα signaling was inhibited. The result confirmed that neutrophil migration rate is reduced when MDMs were incubated with TNFα neutralizing antibody in Z-MDMs (**Figure 7C**, p-value=0.024).

### Suppression of CXCL-8 Through Macrophage Efferocytosis of Apoptotic Cells

**Figure 8:**
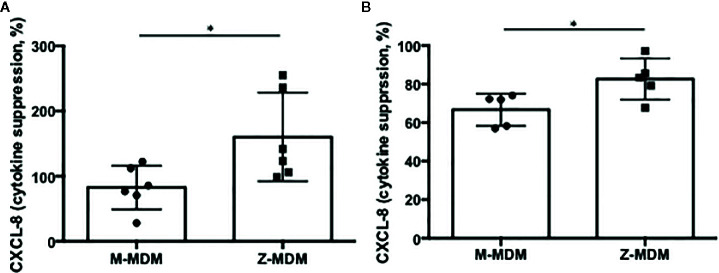
Cytokine suppression in post-efferocytotic macrophages. LPS (10 ng/ml)-stimulated MDMs were incubated with apoptotic **(A)** neutrophils (n=6) or **(B)** Jurkat cells (n=5) for 3 h. Then, the expression level of CXCL-8 was compared between control and post-efferocytotic cells. Data are expressed as mRNA levels relative to control which are set to 100%. *Denotes statistical significance (p-value < 0.05) according to two-tailed Student’s t-test.

Efferocytosis plays a critical role in the resolution of inflammation by preventing the secondary necrosis of dead cells and triggering several anti-inflammatory signalings. Emerging evidence suggests that the expression of inflammatory cytokines is suppressed in post-efferocytotic macrophages (41, 42). We induced inflammatory signaling in MDMs using LPS and examined whether the efferocytosis of apoptotic neutrophils is able to suppress LPS-induced CXCL-8 expression. As shown in **Figure 8A**, macrophage efferocytosis of apoptotic neutrophils suppressed the expression level of CXCL-8 by ~20% in M-MDMs but the cytokine suppression was not observed in Z-MDMs (p-value=0.039). We repeated the assay using apoptotic Jurkat cells (**Figure 8B**). The efferocytosis-mediated suppression in the expression level of CXCL-8 was observed in both M- and Z-MDMs, and the suppressed level was significantly higher in M-MDMs (p-value=0.032). The results imply that AAT plays an important role in macrophage efferocytosis-medicated cytokine suppression and probably in resolving inflammation.

### The Effect of TNFα on the Expression of Efferocytosis-Related Genes

**Figure 9:**
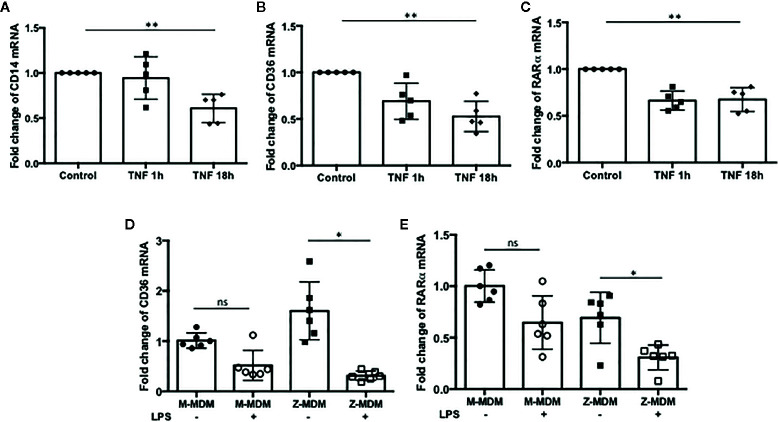
The expression of efferocytosis-related genes regulated by TNFα. MDMs were incubated with TNFα (10 ng/ml) for 1 and 18 h, and the expressions of **(A)** CD14, **(B)** CD36, and **(C)** RARα were examined in the cells (n=5). **Denotes statistical significance (p-value < 0.01) according to one-way ANOVA test. MDMs were incubated with LPS (10 ng/ml) overnight and the relative expression levels of **(D)** CD36, and **(E)** RARα were calculated in comparison to non-treated controls for M-MDMs and Z-MDMs (n=6). *Denotes statistical significance (p-value < 0.05) according to two-tailed Student’s t-test.

Our results showed that LPS-induced TNFα expression inhibits efferocytosis of apoptotic neutrophils by MDMs. It was previously reported that macrophages in a TNFα-rich inflammatory environment are less able to phagocytose apoptotic Jurkat cells (43). We wanted to identify the target molecules that TNFα modulates to inhibit macrophage efferocytosis. A number of genes are involved in the process of efferocytosis. We examined whether TNFα is able to reduce the expression of these genes. MDMs were incubated with TNFα for 1 or 18 h and the expression levels of the genes were examined in the TNFα-treated cells using qRT-PCR. We found that the expressions of CD14, CD36, and RARα are significantly down regulated by TNFα (**Figures 9A** p-value=0.005, **9B** p-value=0.0008, and **9C** p-value=0.0001). These three genes play an important role in macrophage efferocytosis of apoptotic cells; CD14 functions as a bridging molecule that tethers apoptotic cells to macrophages and associates with other molecules within the phagocytic synapse (44). CD36 is essential for macrophage recognition of phosphatidylserine on the surface of apoptotic cells (45). RARα increases apoptotic cell phagocytosis by inducing the expression of phagocytosis-related genes (46). Therefore, TNFα could mediate reduction in efferocytosis of apoptotic cells by inhibiting the expression of CD14, CD36 and RARα in macrophages. We compared the expression level of the genes between LPS-treated M-MDMs and Z-MDMs. LPS treatment reduced the expression of CD36 and RARα in both M- and Z-MDMs, but the reduced level was statistically significant only in Z-MDMs (**Figures 9D** p-value=0.0043 and **9E** p-value=0.0018). The cell surface distribution of CD36 was examined in MDMs. LPS reduced the cell surface level of CD36 in both M- and Z-MDMs, but the reduced level was statistically significant only in Z-MDMs (**Supplementary Figure 3**, p-value=0.014). The expression of CD14 was also reduced by LPS in both M- and Z-MDMs, and it was, on average, reduced more in Z-MDMs than M-MDMs. However, there was no statistical difference in CD14 expression between LPS treated M-MDMs and Z-MDMs (data not shown). We suspect that the combined effect of the reduced expression of CD14, CD36, and RARα lead to the statistically different efferocytosis rate of apoptotic neutrophils between M- and Z-MDMs. However, we could not rule out that there are other molecules, playing a role in reducing the efferocytosis rate of LPS-treated MDMs.

## Discussion

AATD is a genetic disorder leading to emphysema and chronic obstructive pulmonary disease mostly due to a significantly low level of AAT (47); the concentration of circulating AAT is 20–53 μM in normal individuals while it ranges from 3 to 7 μM in AATD individuals with homozygous Z genotype (5). A number of studies aimed to elucidate the pathogenesis of lung diseases associated with AATD and found that alveolar neutrophils are prevalent in AATD individuals (10). It suggests that AAT has an ability to limit excessive neutrophil accumulation to the pulmonary alveolus as well as its primary function of defense against the elastolytic burden in the lower airways posed by various proteases. It has been suggested that AAT has other capabilities that extend beyond its antiprotease role (20). In this study, we demonstrated that AAT has multifaceted abilities to maintain a neutrophil balance in the pulmonary alveolus and consequently a healthy lung. One previous study found that the chemotactic migration rate of PiZZ neutrophils is 2–8 times higher than PiMM neutrophils and extracellularly added AAT reduced neutrophil chemotaxis by binding to CXCL-8 (20). Another study showed that AAT is able to directly bind to LTB4, a potent neutrophil chemoattractant, and consequently inhibits LTB4–BLT1 interaction to reduce neutrophil chemotaxis (48). A common finding of the previous studies is that AAT inhibits neutrophil chemotaxis through its binding to those neutrophil chemotactic factors. Unlike the previous studies, we found that AAT is able to modify the expression of neutrophil chemotactic factors in LPS-stimulated macrophages. Our results showed that the expression level of neutrophil chemotactic factors is significantly higher in LPS-treated Z-MDMs than M-MDMs and that neutrophil migration rate is significantly higher in the conditioned media of Z-MDM culture than that of M-MDM culture. In addition, our data showed that the expression level of CXCL-8 is more significantly suppressed by the efferocytosis of apoptotic cells in M-MDMs than Z-MDMs. Taken together, the results support that AAT is able to inhibit the expression of the neutrophil chemotactic factors in macrophages and thus block excessive neutrophil infiltration to pulmonary alveoli during LPS-mediated inflammation while Z-AAT variant increases the number of alveolar neutrophils in AATD individuals by inducing the expression of the neutrophil chemotactic factors in alveolar macrophages.

Neutrophils are terminally differentiated cells and have a very short life span (49). Immediately after bacterial infection, a number of neutrophils migrate to the infection site. When the recruited neutrophils fulfill their role, neutrophils undergo programmed cell death. Lingering neutrophils exacerbate inflammation and cause tissue injury. Thus neutrophil apoptosis is essential to normal tissue homeostasis. However, inappropriate or premature apoptosis of neutrophils may compromise their function, impairing host defense (50–53). Our data showed that the expression level of TNFα is significantly higher in Z-MDMs than M-MDMs. It was previously reported that TNFα induces neutrophil apoptosis (54). Given that the apoptosis rate of PiZZ neutrophils is two-fold higher than PiMM neutrophils (53) and the expression level of TNFα promoting neutrophil apoptosis is higher in Z-MDMs, Z-AAT variant accelerates neutrophil apoptosis in the pulmonary alveoli of AATD individuals.

Recognition and efferocytosis of apoptotic cells by macrophages is a critical step to resolve inflammation by mediating secretion of anti-inflammatory cytokines TGF-β and IL-10 that inhibit inflammatory response (55, 56). It was previously demonstrated that efferocytosis of apoptotic cells by macrophages could accelerate resolution of LPS-induced lung inflammation in a TGF-β dependent manner (25, 57). Our result showed that LPS inhibited the efferocytosis of apoptotic neutrophils by macrophages and the inhibitory effect of LPS on the efferocytosis was significantly higher in Z-MDMs than M-MDMs, indicating that AAT combats LPS-reduced macrophage efferocytosis. Impaired macrophage efferocytosis could lead to a higher neutrophil infiltration rate to alveoli because prolonged presence of apoptotic neutrophils aggravates inflammation that further increases the expression of neutrophil chemotactic factors in alveolar macrophages. Previous studies on COPD, which is highly associated with Z-AAT variants, found that alveolar macrophages efferocytosis are impaired in COPD patients and suggest that the impaired efferocytosis or phagocytosis by alveolar macrophages could perpetuate an inflammatory response (18, 58–60). Our data showed that LPS-induced efferocytosis impairment was recovered in Z-MDMs when TNFα signaling was inhibited in the cells by TNFα neutralizing antibody. It suggests that TNFα signaling is responsible for the reduced macrophage efferocytosis in LPS-stimulated cells. When MDMs were stimulated with LPS, the expression level of TNFα was significantly higher in Z-MDMs than M-MDMs. It indicates that the clearance of apoptotic neutrophils by alveolar macrophages would be more impaired in AATD individuals by the increased TNFα level during LPS-mediated inflammation. Indeed, TNFα signaling has been proposed to drive immune cell dysfunction causing lung diseases in AATD individuals (47). We attempted to explain how TNFα signaling reduces macrophage efferocytosis and found that the expression levels of CD14, CD36, and RARα were significantly reduced by TNFα in MDMs. This suggests that TNFα signaling inhibits the expression of the three genes to impair macrophage efferocytosis of apoptotic neutrophils. The expression level of the three genes was significantly lower in LPS-treated Z-MDMs and that, at least in part, explains the higher inhibitory effect of LPS on the efferocytosis by Z-MDMs.

The present study highlights the pivotal role of the AAT molecule in modulating the expression of pro-inflammatory cytokines in alveolar macrophages. Upon pro-inflammatory cytokine stimulation, alveolar macrophages produce cytokines and chemokines that attract and activate neutrophils. A high expression level of CXCL-8 has been found in pulmonary diseases, including acute respiratory distress syndrome and idiopathic pulmonary fibrosis (61, 62). TNFα has been suggested to be essential in the pathogenesis of lung diseases associated with AATD (47). The effect of extracellular AAT on regulating TNFα expression and CXCL-8 activity has been well studied. In this study, we focus on examining the effect of intracellular Z-AAT on the expression of CXCL-8 and TNFα in LPS-stimulated MDMs. We show that the level of intracellular Z-AAT is highly increased by LPS treatment, and the expressions of CXCL-8 and TNFα are significantly increased in LPS-treated Z-MDMs. We also show that inhibiting TNFα signaling reduces the expression of CXCL-8 and alleviates the inhibitory effect of LPS on macrophage efferocytosis of apoptotic neutrophils.

**Figure 10:**
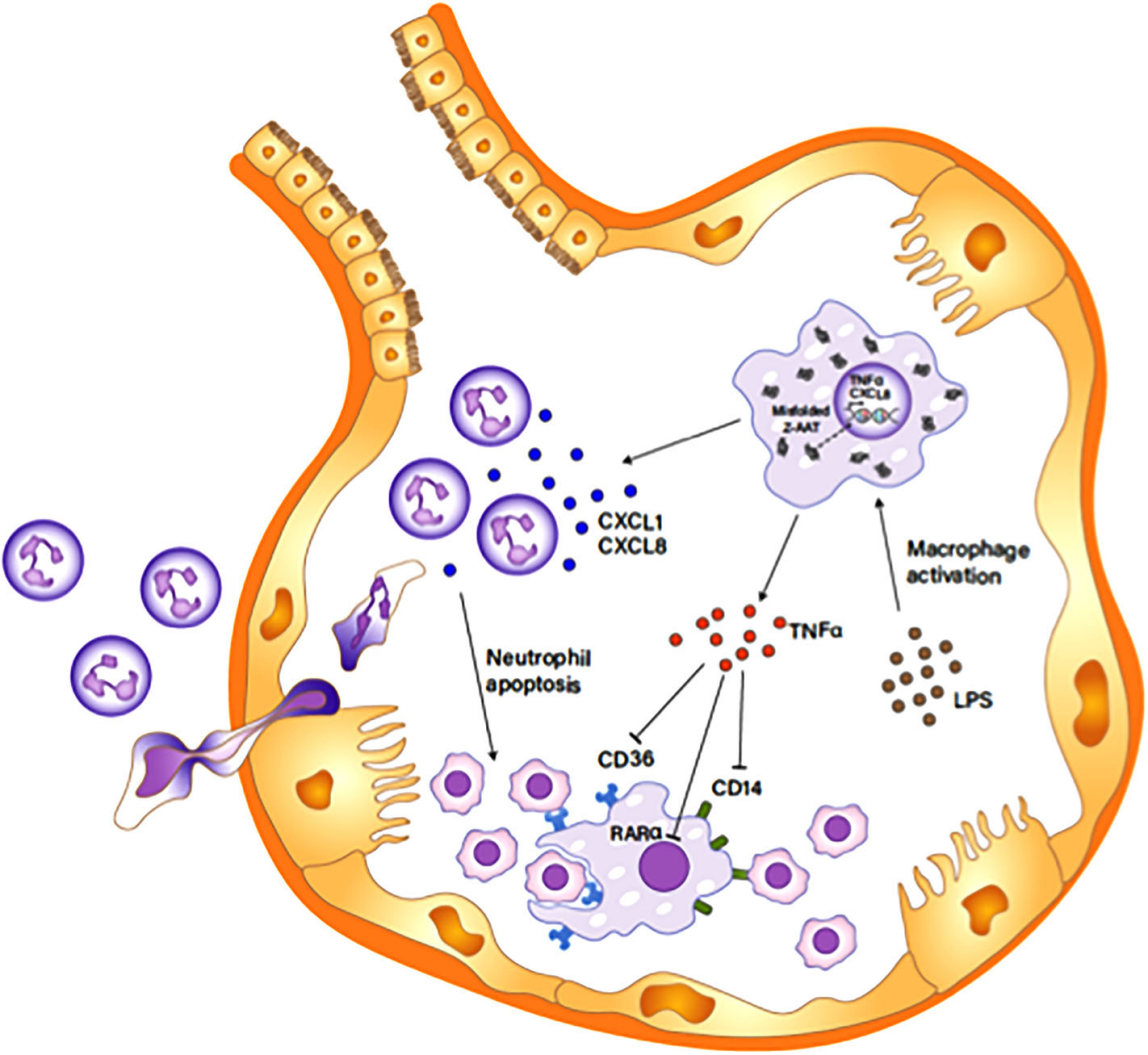
Z-AAT causing excessive neutrophil accumulation in the pulmonary alveolus. Unfolded Z-AAT induces the expressions of CXCL-8 and TNFα in LPS-stimulated macrophages. The increased level of the chemotactic factor accelerates neutrophil infiltration to the pulmonary alveolus. LPS-induced TNFα expression inhibits the expression of CD14, CD36, and RARα that are important in macrophage efferocytosis of apoptotic cells, delaying the clearance of apoptotic neutrophils by macrophages. The impaired clearance of neutrophils aggravates and prolongs the neutrophil influx in the pulmonary alveolus.

In conclusion, Z-AAT accumulation in alveolar macrophages is a main driver for excessive neutrophils in pulmonary alveoli of AATD individuals by inducing the expression of CXCL-8 and TNFα in the cells and, in addition to a low concentration of extracellular AAT, further exacerbates neutrophil burden in the individuals. We propose a mechanism to explain the role of Z-AAT in neutrophil accumulation in pulmonary alveoli of AATD individuals, as depicted in **Figure 10**. The knowledge gained from the present study helps us better understand the multifaceted effect of AAT on regulating neutrophil balance and the underlying mechanisms, which is critical to develop improved therapies to reinforce host defense and attenuate detrimental pulmonary diseases associated with AAT deficiency.

## Data Availability Statement

All datasets presented in this study are included in the article/**Supplementary Material**.

## Ethics Statement

The studies involving human participants were reviewed and approved by University of Florida Institutional Review Board protocol 2015-01051. The patients/participants provided their written informed consent to participate in this study.

## Author Contributions

JL and MB: designed the study, planned the experimental work, and analyzed the data. JL, YL, CM, and KH: performed experimental work and analyzed the data. RO and JW: collected biological samples. JL and MB: wrote the manuscript. YL, KH, CM, RO, and JW: critically reviewed the manuscript. All authors contributed to the article and approved the submitted version.

## Funding

This study was supported by Alpha One Foundation Research Program and Professorship (UFF F007320) and National Center for Advancing Translational Sciences (NCATS-UL1TR001427).

## Conflict of Interest

The authors declare that the research was conducted in the absence of any commercial or financial relationships that could be construed as a potential conflict of interest.

## Supplementary Material

Supplementary Figure 1 Neutrophil isolation and apoptosis. Primary neutrophils were isolated from PiMM volunteers for neutrophil chemotaxis assay. **(A)** The isolated neutrophils were incubated with fluorescent CD16 and CD66b antibodies, and percentage of CD16 and CD66b-positive cells were calculated using Gallios flow cytometer with Kaluza software. **(B)** The apoptotic rate of neutrophils was assessed by flow cytometry with Annexin V/propidium iodide staining.

Supplementary Figure 2 Visualization of neutrophil efferocytosis by MDMs. **M**-MDMs were incubated with red color-labeled apoptotic neutrophils, and the images of phagocytizing MDMs were captured using fluorescent microscope. **(A–C)** indicate the phase contrast of MDMs, red color-labeled apoptotic neutrophils, and MDMs phagocytizing apoptotic neutrophils, respectively.

Supplementary Figure 3 Calculation of neutrophil efferocytosis rate. Z-MDMs were incubated with apoptotic neutrophils in the absence or presence of LPS (10 ng/ml). **(A, C)** show control and LPS-treated cells that were gated on size and granularity, respectively. **(B, D)** show the percentage of control and LPS-treated MDMs phagocytozing apoptotic neutrophils, respectively. In the histogram plot, the first peak indicates non-phagocytosing MDM population and the second peak indicates phagocytosing MDM population.

Supplementary Figure 4 Cell surface distribution of CD36 in MDMs. MDMs were incubated in the absence or presence of LPS (10 ng/ml) overnight. Blue and red fluorescence indicates the nucleus and CD36, respectively. 63x Images were taken using a fluorescence microscope.
