# Supplementary figures and images for: Alpha 1 Antitrypsin-Deficient Macrophages Have Impaired Efferocytosis of Apoptotic Neutrophils
Source: Front Immunol. 2020 Nov 20;11:574410. doi: 10.3389/fimmu.2020.574410 (PMC7714766; doi:10.3389/fimmu.2020.574410)

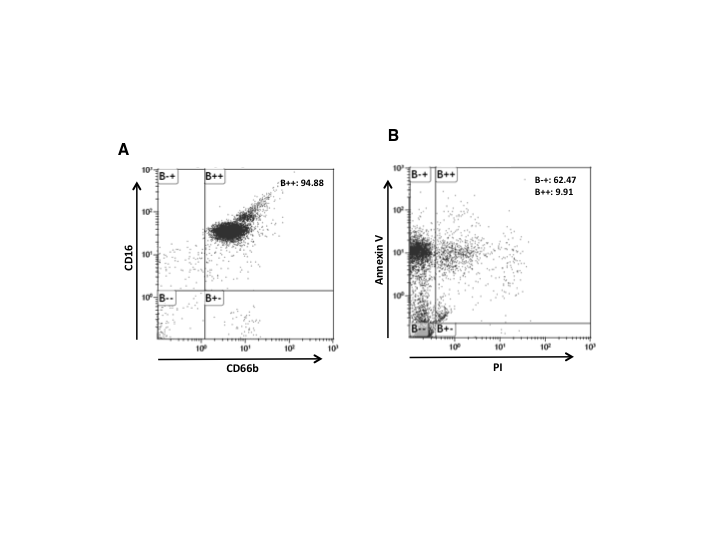

Supplement: Supplementary Figure 1 — Neutrophil isolation and apoptosis. Primary neutrophils were isolated from PiMM volunteers for neutrophil chemotaxis assay. (A) The isolated neutrophils were incubated with fluorescent CD16 and CD66b antibodies, and percentage of CD16 and CD66b-positive cells were calculated using Gallios flow cytometer with Kaluza software. (B) The apoptotic rate of neutrophils was assessed by flow cytometry with Annexin V/propidium iodide staining. [file Image_1.tif]

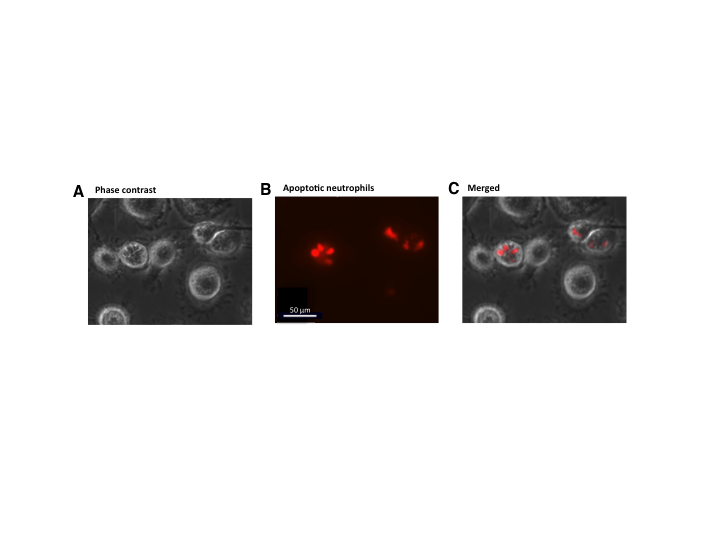

Supplement: Supplementary Figure 2 — Visualization of neutrophil efferocytosis by MDMs. M-MDMs were incubated with red color-labeled apoptotic neutrophils, and the images of phagocytizing MDMs were captured using fluorescent microscope. (A–C) indicate the phase contrast of MDMs, red color-labeled apoptotic neutrophils, and MDMs phagocytizing apoptotic neutrophils, respectively. [file Image_2.tif]

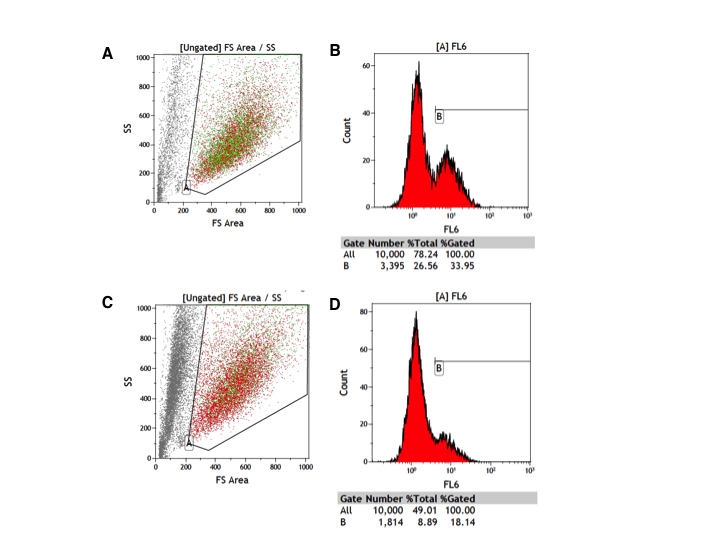

Supplement: Supplementary Figure 3 — Calculation of neutrophil efferocytosis rate. Z-MDMs were incubated with apoptotic neutrophils in the absence or presence of LPS (10 ng/ml). (A, C) show control and LPS-treated cells that were gated on size and granularity, respectively. (B, D) show the percentage of control and LPS-treated MDMs phagocytozing apoptotic neutrophils, respectively. In the histogram plot, the first peak indicates non-phagocytosing MDM population and the second peak indicates phagocytosing MDM population. [file Image_3.tif]

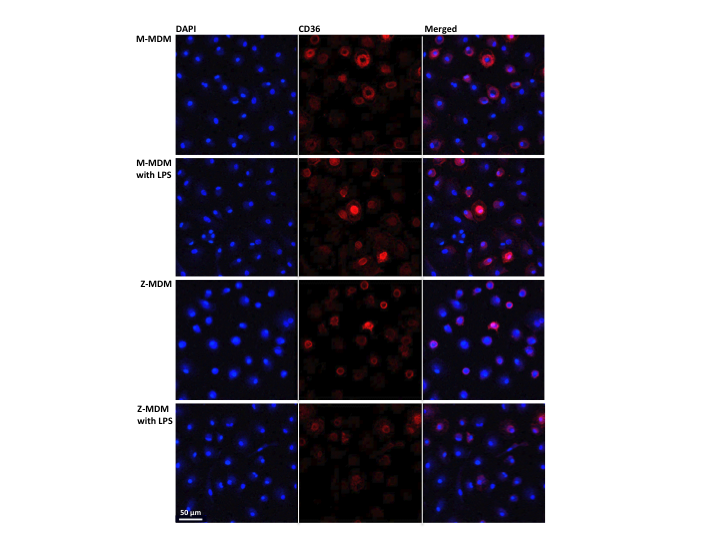

Supplement: Supplementary Figure 4 — Cell surface distribution of CD36 in MDMs. MDMs were incubated in the absence or presence of LPS (10 ng/ml) overnight. Blue and red fluorescence indicates the nucleus and CD36, respectively. 63x Images were taken using a fluorescence microscope. [file Image_4.tif]
